# Hedgehogs and Mustelid Species: Major Carriers of Pathogenic *Leptospira*, a Survey in 28 Animal Species in France (20122015)

**DOI:** 10.1371/journal.pone.0162549

**Published:** 2016-09-28

**Authors:** Florence Ayral, Zoheira Djelouadji, Vincent Raton, Anne-Laure Zilber, Patrick Gasqui, Eva Faure, Florence Baurier, Gwenaël Vourc’h, Angeli Kodjo, Benoît Combes

**Affiliations:** 1 Entente for the Control of Zoonoses, Nancy, France; 2 Université de Lyon, VetAgro Sup, USC 1233, Marcy l’Etoile, France; 3 INRA, UR346 Epidémiologie Animale, Saint Genès Champanelle, France; 4 Fédération Nationale de la Chasse, Issy Les Moulineaux, France; 5 Laboratoire Départemental du Cher, Bourges, France; Universidade Federal de Pelotas, BRAZIL

## Abstract

Human leptospirosis is a zoonotic and potentially fatal disease that has increasingly been reported in both developing and developed countries, including France. However, our understanding of the basic aspects of the epidemiology of this disease, including the source of *Leptospira* serogroup Australis infections in humans and domestic animals, remains incomplete. We investigated the genetic diversity of *Leptospira* in 28 species of wildlife other than rats using variable number tandem repeat (VNTR) and multispacer sequence typing (MST). The DNA of pathogenic *Leptospira* was detected in the kidney tissues of 201 individuals out of 3,738 tested individuals. A wide diversity, including 50 VNTR profiles and 8 MST profiles, was observed. Hedgehogs and mustelid species had the highest risk of being infected (logistic regression, OR = 66.8, CI_95%_ = 30.9–144 and OR = 16.7, CI_95%_ = 8.7–31.8, respectively). Almost all genetic profiles obtained from the hedgehogs were related to *Leptospira interrogans* Australis, suggesting the latter as a host-adapted bacterium, whereas mustelid species were infected by various genotypes, suggesting their interaction with *Leptospira* was different. By providing an inventory of the circulating strains of *Leptospira* and by pointing to hedgehogs as a potential reservoir of *L*. *interrogans* Australis, our study advances current knowledge on *Leptospira* animal carriers, and this information could serve to enhance epidemiological investigations in the future.

## Introduction

*Leptospira* spp. are endemic in many domestic and wild mammals, which may shed the bacteria in their urine [1]. Humans may acquire potentially fatal leptospirosis through direct contact with the urine of infected animals or indirectly through interaction with a urine-contaminated environment. In France, the incidence of leptospirosis was 1/100,000 inhabitants per year in 2014, which was the highest in recent decades [2]; an incidence of 0.5/100,000 inhabitants per year was reported between 2000 and 2010 [3]. Additionally, antibodies against the *Leptospira* serogroup Australis, historically considered uncommon, have recently been implicated in 6% to 18% of infected patients and 43% of leptospirosis cases in livestock, diagnosed in both by the use of a microagglutination test (reference test) [2,4,5]. This change in the disease epidemiology is important for public health and requires a thorough and up-to-date understanding of the disease epidemiology to enhance prevention and preparedness.

Among wildlife species, rodents are considered the primary reservoir hosts for leptospirosis in rural and urban environments [6,7]. Contact with water contaminated with rodent urine is a well-known risk factor for leptospirosis. Rodents worldwide, and more specifically, brown rats in France, are reported to be the main carrier of *Leptospira* serogroup Icterohaemorrhagiae [8]. However, the rat reservoir does not explain the diversity of the serogroups identified in human and domestic animal leptospirosis. Other wildlife species are suspected to have a role in the *Leptospira* transmission cycle because of their frequent seroreactivity to *Leptospira*, which is found in many countries [9–15]. Although *Leptospira* has been detected in the kidneys of ungulates [16–18], little is known about the renal carriage ability over prolonged periods or about the strain circulating in wild animals other than small mammals. Gathering this information in different wildlife species is crucial for a better understanding of the general epidemiology of leptospirosis and for the development of appropriate prevention measures.

The objectives of this study were (1) to describe the *Leptospira* strains circulating in wildlife other than rats using variable number tandem repeat (VNTR) and multispacer sequence typing (MST), (2) to identify the animal species with the highest prevalence of leptospiral renal carriage, (3) to identify the animal species that would predominantly carry *Leptospira* related to the serogroup Australis, and (4) to assess the potential role of wildlife species in maintaining *Leptospira* in France. Finally, we addressed the results from a combination of approaches, while considering the implications for infection risk in humans and domestic animals.

## Methods

### Sample collection

The authors assert that no animals were killed for the purposes of this study and that all procedures contributing to this work complied with the ethical standards of the relevant national and European regulations on the care and use of animals (Directive 2010/63/EC).

A survey was conducted in 30 “départements” (*i*.*e*., administrative districts) in the mainland of France, which were included based on an agreement with the public authorities. The study area covered 175,000 km and included a population of 21 million people. The sample design was standardized at the département level as follows: 141 individual wild animals were collected from each département to produce an appropriate sample size (*i*.*e*., 95% confidence to detect at least 1 infected individual if the prevalence of infection in the overall wildlife population is greater than 2%). To ensure homogeneous sampling among départements, the collection of at least 10 individuals from each of the following preponderant species was recommended: red deer (*Cervus elaphus*), roe deer (*Capreolus capreolus*), wild boar (*Sus scrofa*), fox (*Vulpes vulpes*), stone marten (*Martes foina*), pine marten (*Martes martes*), hare (*Lepus europaeus*), and rabbit (*Oryctolagus cuniculus*). The remaining individuals could belong to any wild mammal species, except for small mammals such as rats (*Rattus* spp.), bats (*Chiroptera* spp.), coypu (*Myocastor coypus*), and muskrats (*Ondatra zibethicus*), for which leptospiral renal carriage has been documented in France [19,20].

Hunting, population control, and animals found dead were used as sources of the collected individuals. Only individuals who had died within the previous 48 hours or bodies without signs of deterioration were included to prevent any bias related to PCR inhibitors. The field researchers were trained to sample the dead animals following predetermined guidelines. The methods of collection were performed regardless of the time of year, except for the collection of hunted animals, which were collected during hunting campaigns (*i*.*e*., from September to February between 2012 and 2015).

Kidney tissues were removed immediately after death or after the discovery of accidentally killed animals and were transported to the laboratory, where the tissues were frozen at -20°C until further analysis.

### Molecular investigations

**Fig 1:**
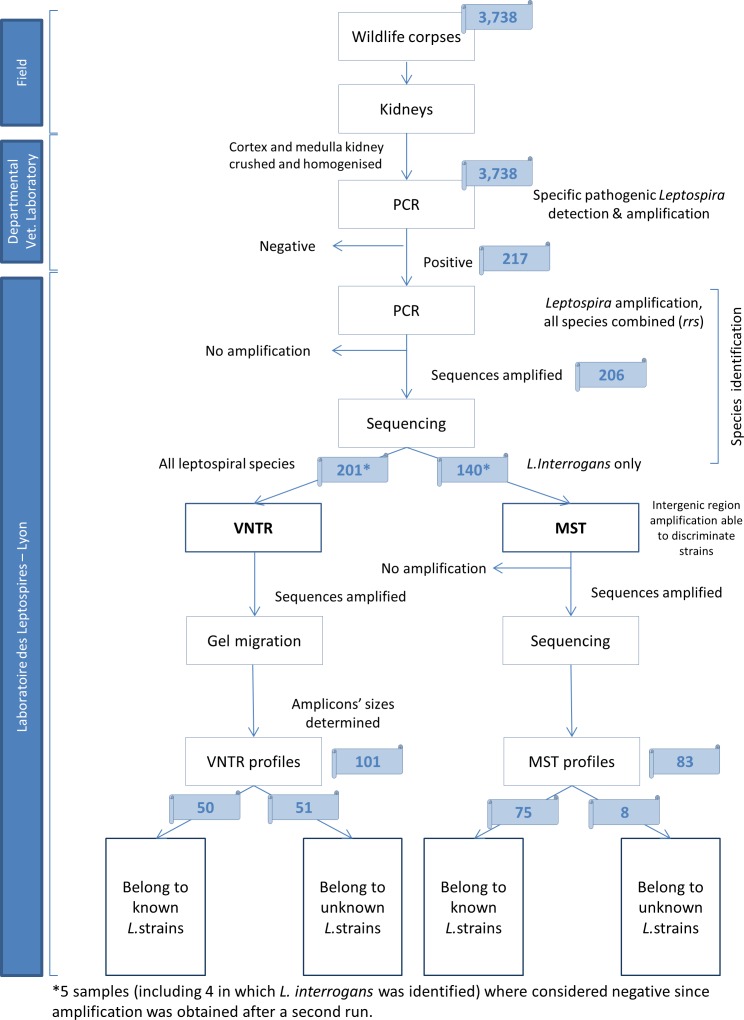
Flow chart of the laboratory analysis and sample counting.

Details of the laboratory analyses are provided below and summarized in Fig 1.

One-fourth of each kidney was homogenized aseptically using a syringe. A small amount of this crushed kidney (approximately 25 mg) was incubated with 180 μl of ATL Buffer and 25 μl of proteinase K (QIAamp, Qiagen, Courtaboeuf, France) for 3 hours. After protein digestion, the DNA was extracted from 200 μl of lysed tissue using a Nucleospin Tissue kit (QIAamp, Qiagen, Courtaboeuf, France) according to the manufacturer’s instructions. All DNA samples were stored at -20°C.

The extent of *Leptospira* colonization of the kidney was assessed using a specific pathogenic *Leptospira* TaqMan real-time PCR kit (TaqVet PathoLept kit, Lifetech, Lissieu, France) according to the manufacturer’s instructions and PCR mix without the target DNA was included as a negative control. The removal of PCR inhibitors in the samples was confirmed using an internal control called IPC (Internal Positive Control). Correct amplification of the IPC at a cycle threshold (Ct) of 26 following calibration was required for validation of *Leptospira* amplification. Specimens with a Ct of less than 45 cycles were considered positive as suggested by the manufacturer’s instructions. This threshold is higher than the 40-cycle threshold usually used for a positive sample and may increase the false-positive rate. Therefore, DNA characterization was performed to provide further evidence of pathogenic *Leptospira* occurrence and had served as a second control for positive samples.

As the first step of DNA characterization, the *rrs* (16S) gene was amplified by PCR using HotStarTaq DNA Polymerase (Qiagen) under standard conditions and with previously described primers [21]. The *Leptospira* species in the samples were identified by analyzing the *rrs* (16S) sequences using NCBI nucleotide BLAST software (http://blast.ncbi.nlm.nih.gov).

As a second step, VNTR and MST typing was performed, and serovar identities were deduced from the VNTR and MST profiles obtained, according to previously published frameworks [22,23].

### Descriptive analysis

The primary outcome variable was *Leptospira* infection status (positive *vs*. negative). Given that *rrs* (16S) gene sequencing is highly specific for determining *Leptospira* species, individuals were considered infected if the typing result was consistent with pathogenic *Leptospira* genospecies. The spatial distribution of the infectious status of the individuals sampled across the study areas was visualized in ArcGIS version 9.3 (ESRI, Redland, CA, USA).

To study the distribution of *Leptospira* genotypes in populations, the results were interpreted as follows: when both a VNTR profile and a MST profile were related to the same individual, the serovar deduced from the MST profile was preferred because MST is often more discriminating than VNTR [22].

The prevalence of infection was defined as the proportion of infected *vs*. uninfected individuals in the sampled populations. To improve the clarity and flow of the results, the animal species were classified into the following groups: large carnivores, mustelids, erinaceomorphs, lagomorphs, rodents, and ungulates. The prevalence of infection was then calculated for each animal species and each animal group. The confidence intervals for infection prevalence were calculated using the Clopper-Pearson method [24].

### Statistical analysis

Statistical modeling was performed to assess which animal group was the best predictor of leptospiral renal carriage in wildlife. A GLMM_1_ was used to examine the relation between *Leptospira* carriage and the animal groups. Hierarchical data (*i*.*e*., animal species nested in groups) were considered to account for the variation in the prevalence among animal species within the groups and the variation in the number of animal species included in the groups. Additionally, the random effect of the département was used to control for the potential effects of clustering. The ungulates were used as the reference group because they have been previously implicated as leptospiral reservoirs [16,18].

All statistical analyses were conducted using R software, version 3.0.1 (R Development Core Team [2013], R Foundation for Statistical Computing, Vienna, Austria). The GLMMs were performed using the “glmer” function of the {lme4} Package.

## Results

### Population description

A total of 3,738 individuals from 28 animal species were tested by PCR for pathogenic *Leptospira*. Most of the individuals (39%, n = 1461) were ungulates, 1,411 (38%) were carnivores, 683 (17%) were lagomorphs, 112 (3%) were erinaceomorphs (*i*.*e*., hedgehogs), and 81 (2%) were rodents other than rats.

Hunting was the primary source of samples (39%, n = 1467), followed by accidental death (23%, n = 852) and population control measures (8%, n = 319).

### Leptospiral carriage

**Fig 2:**
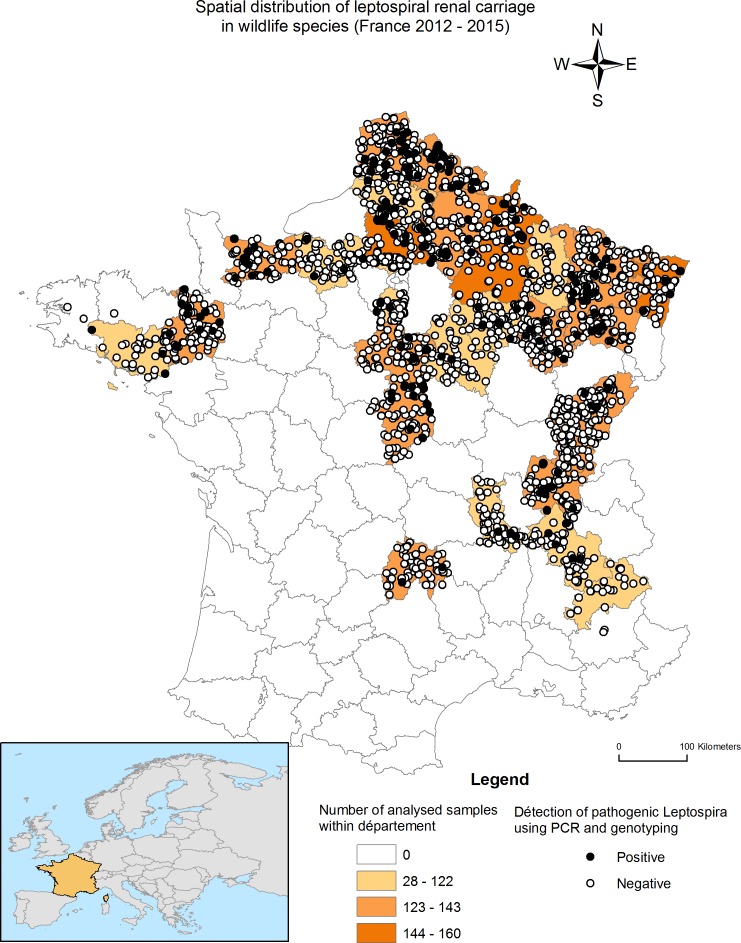
The spatial distribution of infectious status for pathogenic *Leptospira* among wildlife in the study area.

**Fig 3:**
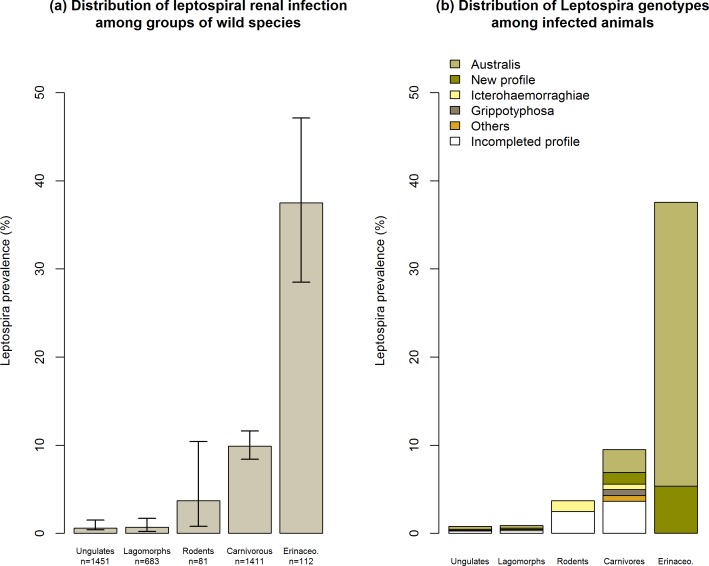
(a) Distribution of *Leptospira* prevalence (%) among the groups of wild animal species and (b) distribution of *Leptospira* genotypes among infected animals.

Based on *rrs* (16S) gene typing, 201 individuals were found infected with pathogenic *Leptospira*. The overall prevalence of leptospiral renal carriage in the sampled population was 5.4% (CI_95%_ = 4.7–6.1%), and the infected individuals were widely distributed throughout the study area (Fig 2). Variations in infection prevalence by groups of animal species were observed (Fig 3A), with values ranging from 0.8% to 37.5%. Considering the animal species (Table 1), the prevalence was greatest in hedgehogs (37.5% CI_95%_ = 28.5–47.1%), followed by weasels (20.6% CI_95%_ = 8.7–38%) and pine martens (15.4% CI_95%_ = 10.4–21.6%).

**Table 1:** The baseline characteristics and prevalence of *Leptospira* kidney carriage among the wildlife species sampled.

		* *	*Leptospira* PCR status			
Groups	Animal species	* *	Total no.	No. pos.	Prev. (%)	95% CI
		* *				
**Carnivores**		* *	** **	** **	** **	** **
**Large carnivores**	** **	*** ***	**545**	**32**	**5.9**	**4–8.2**
	European wild cat	*Felis silvestris silvestris*	30	2	6.7	0.8–22
	Feral cat	*Felis silvestris catus*	88	4	4.5	1.2–11.2
	Fox	*Vulpes vulpes*	362	22	6.1	3.8–9.1
	Lynx	*Lynx*	7	0	0	0–41
	Raccoon	*Procyon lotor*	52	4	7.7	2.1–18.5
	Western wolf	*Canis lupus*	6	0	0	0–46
		* *				
**Mustelids**	** **	*** ***	**866**	**106**	**12.2**	**10.1–14.6**
	Badger	*Meles meles*	316	26	8.2	5.4–11.8
	Stone marten	*Martes foina*	205	29	14.1	9.7–19.7
	European pine marten	*Martes martes*	175	27	15.4	10.4–21.6
	European polecat	*Mustela putorius*	107	15	15.0	8.1–22.1
	Least weasel	*Mustela nivalis*	34	7	20.6	8.7–38
	European otter	*Lutra lutra*	5	0	0	0–52
	Stoat	*Mustela ermine*	24	2	8.3	1.0–27
		* *				
**Erinaceomorpha**	** **	*** ***	**112**	**42**	**37.5**	**28.5–47.1**
	Hedgehog	*Erinaceus europaeus*	112	42	37.5	28.5–47.1
		* *				
**Lagomorphs**	** **	*** ***	**683**	**6**	**0.9**	**0.3–1.9**
	European hare	*Lepus europaeus*	367	5	1.4	0.4–3.1
	European rabbit	*Oryctolagus cuniculus*	314	1	0.3	0.01–1.8
	Mountain hare	*Lepus timidus*	2	0	0	0–84
		* *				
**Rodents**	** **	*** ***	**81**	**3**	**3.7**	**0.8–10.4**
	European beaver	*Castor fiber*	9	3	33.3	7.5–70
	Edible dormouse	*Glis glis*	3	0	0	0–71
	Marmot	*Marmota*	3	0	0	0–71
	Squirrel	*Spermophilus*	66	0	0	0–5.4
		* *				
**Ungulates**	** **	*** ***	**1451**	**12**	**0.8**	**0.4–1.4**
	Alpine ibex	*Capra ibex*	4	0	0	0–60.2
	Chamois	*Rupicapra rupicapra*	64	0	0	0–5.6
	Fallow deer	*Dama dama*	14	0	0	0–23.1
	Red deer	*Cervus elaphus*	332	1	0.3	0.01–1.7
	Roe deer	*Capreolus capreolus*	498	7	1.4	0.6–2.9
	Mouflon	*Ovis*	32	0	0	0–11
	Wild boar	*Sus scrofa*	507	4	0.8	0.2–2.0
**Total**	** **	*** ***	**3738**	**201**	**5.4**	**4.7–6.1**

### *Leptospira* genotypes

Three *Leptospira* genospecies were identified, including *L*. *interrogans* (n = 140), *L*. *kirschneri* (n = 37) and *L*. *borgpetersenii* (n = 25). In addition, DNA from two *Leptospira* species (*L*. *kirschneri* and *L*. *borgpetersenii*) was extracted from a stone marten sample. From the 201 leptospiral DNA samples extracted, VNTR profiles were obtained for 101 individuals, including 14 profiles previously reported in reference strains and 36 unreported ones (Table 2). From the 140 *L*. *interrogans* DNA samples extracted, MST profiles were obtained for 83 individuals, including 6 profiles previously reported in reference strains and 2 unreported ones (Table 3).

**Table 2:** List of the variable number tandem repeat (VNTR) profiles and the serogroups and serovars deduced from the analysis of leptospiral DNA.

Results for locus						
VNTR-4	VNTR-LB4	VNTR-LB5	species	serogroups	serovars	No. of individuals
1	10	9	*L*. *interrogans*	Australis	Bratislava	19
1	7	13	*L*. *interrogans*	Australis	Fugis	1
3	14	7	*L*. *interrogans*	Autumnalis	Mooris	1
1	10	7	*L*. *interrogans*	Djasiman	Gurungi	8
0	2	9	*L*. *interrogans*	Grippotyphosa	Valbuzzi	1
23	0	2	*L*. *interrogans*	Hebdomadis	Kremastos	1
2	1	7	*L*. *interrogans*	Icterohaemorrhagiae	Icterohaemorrhagiae or Copenhageni	7
3	10	7	*L*. *interrogans*	Pyrogenes	Camlo	1
3	2	11	*L*. *interrogans*	Sejroe	Wolffi-romanica	1
0	10	10	*L*. *interrogans*	*unreported profile*		1
1	10	12	*L*. *interrogans*	*unreported profile*		1
1	9	11	*L*. *interrogans*	*unreported profile*		2
1	10	8	*L*. *interrogans*	*unreported profile*		2
1	9	8	*L*. *interrogans*	*unreported profile*		1
1	8	9	*L*. *interrogans*	*unreported profile*		1
1	9	7	*L*. *interrogans*	*unreported profile*		1
1	9	9	*L*. *interrogans*	*unreported profile*		1
1	8	1	*L*. *interrogans*	*unreported profile*		1
2	1	8	*L*. *interrogans*	*unreported profile*		1
2	3	7	*L*. *interrogans*	*unreported profile*		1
2	10	10	*L*. *interrogans*	*unreported profile*		1
2	10	2	*L*. *interrogans*	*unreported profile*		1
2	10	2	*L*. *interrogans*	*unreported profile*		1
2	9	2	*L*. *interrogans*	*unreported profile*		1
2	11	9	*L*. *interrogans*	*unreported profile*		1
2	9	2	*L*. *interrogans*	*unreported profile*		1
2	9	9	*L*. *interrogans*	*unreported profile*		1
2	9	10	*L*. *interrogans*	*unreported profile*		1
2	2	8	*L*. *interrogans*	*unreported profile*		1
3	10	2	*L*. *interrogans*	*unreported profile*		1
3	9	2	*L*. *interrogans*	*unreported profile*		1
3	9	6	*L*. *interrogans*	*unreported profile*		1
3	11	2	*L*. *interrogans*	*unreported profile*		3
3	6	2	*L*. *interrogans*	*unreported profile*		6
3	8	3	*L*. *interrogans*	*unreported profile*		1
3	10	2	*L*. *interrogans*	*unreported profile*		2
4	10	2	*L*. *interrogans*	*unreported profile*		4
5	10	2	*L*. *interrogans*	*unreported profile*		1
*Unsuccessful amplification*			*L*. *interrogans*			58
1	4	6	*L*. *borgpetersenii*	Ballum	Castellonis	1
2	6	5	*L*. *borgpetersenii*	Pyrogenes	Hamptoni	1
2	6	7	*L*. *borgpetersenii*	*unreported profile*		1
7	6	7	*L*. *borgpetersenii*	*unreported profile*		1
12	0	0	*L*. *borgpetersenii*	*unreported profile*		1
*Unsuccessful amplification*			*L*. *borgpetersenii*			20
0	1	12	*L*. *kirschneri*	Icterohaemorrhagiae	Ndambari	2
2	2	12	*L*. *kirschneri*	Grippotyphosa	Vanderhoedeni	3
0	6	2	*L*. *kirschneri*	Grippotyphosa	Valbuzzi	4
0	1	0	*L*. *kirschneri*	*unreported profile*		1
0	2	2	*L*. *kirschneri*	*unreported profile*		1
0	2	5	*L*. *kirschneri*	*unreported profile*		2
0	2	11	*L*. *kirschneri*	*unreported profile*		1
*Unsuccessful amplification*			*L*. *kirschneri*			23

**Table 3:** List of the multispacer sequence typing profiles (MST) and the serogroups and serovars deduced from the analysis of leptospiral DNA in individuals infected with *L*. *interrogans*.

No. of genotypes by MST profiles						
MST1	MST3	MST9	*L*. *interrogans* serogroups	*L*. *interrogans* serovars	Strains	No. of individuals
6	3	3	Australis	Australis		1
5	11	6	Australis	Muenchen/Jalna/Bratislava		67
13	17	10	Grippotyphosa	Valbuzzi		1
4	10	3	Icterohaemorrhagiae	Icterohaemorrhagiae	CHU Réunion	3
4	6	3	Icterohaemorrhagiae	Copenhageni	M20/Wijinberg	1
4	7	3	Icterohaemorrhagiae	Icterohaemorrhagiae	R1	2
4	20*	3	*unreported profile*			1
6	11	6	*unreported profile*			7
6	18*	-	*incomplete*			1
-	11	12	*incomplete*			1
-	11	6	*incomplete*			1
6	-	-	*incomplete*			2
-	11	-	*incomplete*			13
-	-	-	*Unsuccessful amplification*			39

* Genotypes newly identified in this study; genotypes 18 and 20 correspond to GenBank accession numbers KT923088 and KT923089, respectively.

All three *Leptospira* species (*L*. *interrogans*, *L*. *borgpetersenii* and *L*. *kirschneri*) were found in carnivores (large carnivores and mustelids) and ungulates, whereas renal carriage was limited to *L*. *interrogans* in lagomorphs and rodents. Among the infected erinaceomorphs (n = 42), 41 individuals carried *L*. *interrogans*, and one carried *L*. *borgpetersenii*. The spatial distribution of the *Leptospira* species did not show any specific pattern (S1 and S2 Figs).

An analysis of the genotypes (Fig 3B) revealed a variety of profiles in most of the animal groups. In contrast, the erinaceomorphs were mainly infected with an *L*. *interrogans* profile related to the Bratislava, Jalna or Muenchen serovar (3 serovars that are indistinguishable using MST) and by leptospires, whose genotype was closely related (one nucleotide variation) to the former profile.

### Statistical analysis

The generalized linear mixed model (GLMM_1_) analysis revealed that the odds of *Leptospira* infection were significantly greater in large carnivores, mustelids, erinaceomorphs, and rodents compared to ungulates (Table 4). The erinaceomorphs, including hedgehogs (OR = 66.8, CI = 30.9–144), had the highest odds of *Leptospira* infection, followed by the mustelid species (OR = 16.7, CI = 8.7–31.8).

**Table 4:** The baseline characteristics, prevalence of *Leptospira* kidney carriage, and odds ratio for testing positive for pathogenic *Leptospira* among the wildlife species sampled.

	*Leptospira* PCR status		* *	* *	* *
Groups of animal species	No. neg.	No. pos.	OR	95% CI	p-value
					
Carnivores					
Large carnivores	513	32	7.3	[3.5–15.0]	<0.001
					
Mustelids	760	106	16.7	[8.7–31.8]	<0.001
					
Erinaceomorpha	70	42	66.8	[30.9–144]	<0.001
					
Lagomorphs	677	6	1.0	[0.4–2.9]	0.936
					
Rodents	78	3	4.8	[1.2–18.1]	0.021
					
Ungulates	1439	12	ref	ref	-
Total	3537	201			

## Discussion

This study investigated *Leptospira* strains circulating in 28 wildlife species other than rats using DNA characterization tools. Our results indicate that hedgehogs and mustelid species are substantial leptospiral carriers in France. Interestingly, the hedgehogs’ kidneys were mainly colonized by *L*. *interrogans* genotypes related to the Australis serogroup, whereas the kidneys of carnivores, and more specifically, mustelid species, were mainly colonized by a variety of *Leptospira* genotypes. This distribution suggests that hedgehogs potentially act as a reservoir for the serogroup Australis, whereas the carnivores would have a different role in leptospiral persistence.

### *Leptospira* detection and identification

Among 3,738 individuals, the overall prevalence of *Leptospira* renal carriage was 5.4% of individuals, with the maximum of 37.5% in erinaceomorphs (*i*.*e*., hedgehogs) and the minimum of 0.8% in ungulates. These results show that pathogenic leptospires are found with a heterogeneous distribution in many animal species other than rodents.

This prevalence is an underestimate because *rrs* gene amplification was not observed for 11 PCR-positive samples. Of these 11 samples, 7 were below the cycle threshold of 40 usually considered for a positive result. The absence of amplification is most likely related to DNA lability during transport. The remaining four samples were above the cycle threshold of 40 and may be false-positive results because the test can be less specific under such conditions. However, using a 45-cycle threshold for a positive sample decreased false-negative samples. Among the 201 PCR-positive samples in which amplification was obtained, 7 were above the cycle threshold of 40, suggesting that by considering a threshold of 40, some positive samples may be missed. A combination of PCR and *rrs* gene typing should be considered in future surveys for improved prevalence estimation. In addition, false negatives can still be obtained if PCR is used to identify uncharacterized pathogenic strains [25] or if the bacteria have aggregated in an unsampled part of the kidney.

In ungulates and foxes, we observed prevalences of 0.8% and 6.1%, respectively, whereas no seroconversion was observed in the 1980s in 16 départements that were included in the present study [26]. This discrepancy emphasizes the limitations of serology for *Leptospira* surveys because serology is not sensitive in many animal species [27]. Therefore, serology should not be used without complementary molecular analysis in animal surveys.

The prevalence of 0.8% in wild boars cannot be compared to previous results in Europe as most of the surveys performed used serology. In the latter, the *Leptospira* seroprevalence varied from 10% to 32% [14,15]. The variation observed between exposure and infection rates could be explained by the transitory infection in wild boars. Further investigations are needed to clarify this point.

With the identification of 50 VNTR profiles and 8 MST profiles, our study reflects the wide diversity of *Leptospira* genotypes circulating in wildlife. Although some of the isolated DNA could not be amplified, most likely because of low DNA concentrations, this degree of diversity was previously unreported. The inventory of *Leptospira* strains obtained in our wildlife sample could be used for the purpose of source tracking in the future. Indeed, many of the VNTR and MST profiles found in our survey were identical to those of reference strains that have been isolated from humans in various areas (*e*.*g*., *L*. *interrogans* Djasiman Gurungi and *L*. *interrogans* Grippotyphosa Valbuzzi). This finding indicates that the genotypes of the strains circulating in wildlife and humans are closely related. If a patient were infected in a specified area with one of the VNTR or MST profiles described in our study, it would be possible to speculate that the transmission occurred via local wildlife populations. For this purpose, the development of an inventory of strains circulating in humans is now important to allow comparisons with strains of animal origin.

### *Leptospira* related to the serogroup Australis and host carriage

The predominant VNTR and MST profiles found in our study were related to *Leptospira* serogroup Australis, which has recently been implicated in human and livestock diseases [2,4,5]. The detection of a single MST profile related to the serogroup Australis in 41 of the 42 infected hedgehogs throughout the three years of the study suggests the selective carriage of the specified *Leptospira* strain over the long term. In addition, our study reveals that the risk of renal carriage was significantly greater in hedgehogs (OR = 66.8, CI = 30.9–144). The odds ratio was obtained by controlling the potential effect of clustering; thus, high risk was observed regardless of the department and was not due to spatial clusters. Therefore, hedgehogs appear to be a maintenance population, as defined by Viana et al. [28], for a *Leptospira* strain related to serogroup Australis. Studies conducted several decades ago reported the hedgehog as a carrier of this serogroup in Scotland, Italy, Denmark, the Netherlands, and France using serology and/or bacteriology [29–33]; thus, our study provides more evidence of this specific carriage over large areas and time spans. Finally, the predominance of *Leptospira* related to the serogroup Australis was not observed in any of the remaining wild animal species. Although some wildlife species were not investigated here, with 28 animal species, our study is the largest to date on *Leptospira* carriage and includes the most abundant wildlife species found in France. Our results suggest that the hedgehog is one of the predominant wildlife species that could serve as a source of leptospirosis in humans and domestic animals infected with *Leptospira* serogroup Australis.

### Wildlife maintenance community

The potential exists for connections between wildlife species through direct contact or contact with a common source of *Leptospira* (infectious hosts or environments). In addition, the different distribution patterns of *Leptospira* genotypes in hedgehogs (one predominant VNTR or MST profile) *vs*. carnivores (a variety of VNTR or MST profiles) suggests the different roles of these animal groups in the epidemiological cycle of *Leptospira*. Carnivores might be exposed through their environment and their diet, as many small mammals, often carriers of the various *Leptospira*, can be eaten by them. Our results show that badgers, stone martens, pine martens, and foxes were infected by the various genotypes and could play the role of a sentinel, reflecting the strain circulating in a specified environment, or they may act as the maintenance community, as defined by Viana et al. [28]. For instance, the brown rat is reported to be the primary host of *L*. *interrogans* related to the serogroup Icterohaemorrhagiae, which is responsible for the most severe forms of the disease in humans. In our study, a limited amount of individuals (n = 10), mainly carnivorous (n = 7), were identified as infected with this strain, which suggests that wildlife species other than rats are sporadic hosts. From a public health perspective, information on the possible role of these species as a sentinel and/or maintenance community is critical to properly clarify our understanding of *Leptospira* transmission in humans. Therefore, further studies on the ability of potentially connected wildlife species to spread and maintain *Leptospira* for a prolonged period are required. The application of molecular epidemiology tools could provide substantial information by generating connections between the *Leptospira* DNA samples collected from wildlife.

### *Leptospira* exposure risk of populations

Our study confirms that many wildlife species are leptospiral carriers and may be responsible for environmental contamination. As leptospirosis is re-emerging in humans worldwide [34,35], these findings highlight the importance of leptospiral surveillance in wildlife beyond rodent species.

In addition, our study reports the presence of *Leptospira* in commonly hunted animal species such as wild boars, deer and hares. These observations indicate a risk of leptospirosis for hunters, gamekeepers, and people who deal with the processing of wild meat. In France (2008), 5% (n = 3/62) of human leptospirosis has been estimated to be related to hunting and game keeping, whereas 30% is due to occupational exposure [36]. Elsewhere, being a hunter or a forest worker has been considered one of the main risk factors for leptospirosis [37,38]. Therefore, in France, people are more likely to be exposed from contact with contaminated environment or water (*e*.*g*., hikers, campers, and kayakers) than from handling infected carcasses when hunting. However, the risk of *Leptospira* exposure is present in hunters, and they should be warned that red deer, roe deer, wild boars, hares, and rabbits may be infected by *Leptospira* and take appropriate measures, such as handling the bodies with caution (using gloves) and paying particular attention to avoid contact with biological fluids that may be infected. In addition, people in contact with wild grazing animals, even in urban areas and public gardens where hedgehogs can live or pass, should avoid contact with stagnant water through ingestion or through exposure of the mucosa or abraded skin.

## Supporting Information

S1 Fig Spatial distribution of *Leptospira* species among the hedgehogs tested. (TIF)

S2 Fig Spatial distribution of *Leptospira* species among the carnivores tested. (TIF)

## References

[1] Adler B, de la Peña Moctezuma A. Leptospira and leptospirosis 1st ed. Heidelberg: Springer-Verlag; 2010 10.1016/j.vetmic.2009.03.012

[2] Centre National de Référence de la Leptospirose. Rapport annuel d’activité. Institut Pasteur. 2014. Available at: http://www.pasteur.fr/fr/sante/centres-nationaux-reference/les-cnr/leptospirose/rapports-d-activite. Accessed 6 April 2016.

[3] Dupouey J, Faucher B, Edouard S, Richet H, Kodjo A, Drancourt M, et al. Human leptospirosis: an emerging risk in Europe? Comp Immunol Microbiol Infect Dis. 2014;37: 77–83. 10.1016/j.cimid.2013.12.002 24388481

[4] Centre National de Référence de la Leptospirose. Rapport annuel d’activité. Institut Pasteur. 2013. Available at: http://www.pasteur.fr/fr/sante/centres-nationaux-reference/les-cnr/leptospirose/rapports-d-activite. Accessed 12 September 2015.

[5] Ayral FC, Bicout DJ, Pereira H, Artois M, Kodjo A. Distribution of Leptospira serogroups in cattle herds and dogs in France. Am J Trop Med Hyg. 2014;91: 756–759. 10.4269/ajtmh.13-0416 25092816 PMC4183399

[6] Bharti AR, Nally JE, Ricaldi JN, Matthias MA, Diaz MM, Lovett MA, et al. Leptospirosis: a zoonotic disease of global importance. Lancet Infect Dis. 2003;3: 757–771. 10.1016/S1473-3099(03)00830-2 14652202

[7] Vinetz JM, Glass GE, Flexner CE, Mueller P, Kaslow DC. Sporadic urban leptospirosis. Ann Intern Med. 1996;125: 794–798. 10.7326/0003-4819-125-10-199611150-00002 8928985

[8] Ayral F, Zilber A-L, Bicout DJ, Kodjo A, Artois M, Djelouadji Z. Distribution of Leptospira interrogans by multispacer sequence typing in urban Norway rats (Rattus norvegicus): a survey in France in 2011–2013. PLOS ONE. 2015;10: e0139604 10.1371/journal.pone.0139604 26447693 PMC4598087

[9] Andreoli E, Radaelli E, Bertoletti I, Bianchi A, Scanziani E, Tagliabue S, et al. Leptospira spp. Infection in wild ruminants: a survey in Central Italian Alps. Vet Ital. 2014;50: 285–291. 10.12834/VetIt.1309.06 25546066

[10] Espí A, Prieto JM, Alzaga V. Leptospiral antibodies in Iberian red deer (Cervus elaphus hispanicus), fallow deer (Dama dama) and European wild boar (Sus scrofa) in Asturias, Northern Spain. Vet J. 2010;183: 226–227. 10.1016/j.tvjl.2008.10.003 19019711

[11] Montagnaro S, Sasso S, De Martino L, Longo M, Iovane V, Ghiurmino G, et al. Prevalence of antibodies to selected viral and bacterial pathogens in wild boar (Sus scrofa) in Campania region, Italy. J Wildl Dis. 2010;46: 316–319. 10.7589/0090-3558-46.1.316 20090052

[12] Millán J, Candela MG, López-Bao JV, Pereira M, Jiménez MA, León-Vizcaíno L. Leptospirosis in wild and domestic carnivores in natural areas in Andalusia, Spain. Vector Borne Zoonotic Dis. 2009;9: 549–554. 10.1089/vbz.2008.0081 18973450

[13] Slavica A, Cvetnić Ž, Milas Z, Janicki Z, Turk N, Konjević D, et al. Incidence of leptospiral antibodies in different game species over a 10-year period (1996–2005) in Croatia. Eur J Wildl Res. 2008;54: 305–311. 10.1007/s10344-007-0150-y

[14] Slavica A, Cvetnić Z, Konjević D, Janicki Z, Severin K, Dežđek D, et al Detection of Leptospira spp. serovars in wild boars (Sus scrofa) from Continental Croatia. Vet Arh. 2010;80: 247–257.

[15] Żmudzki J, Jabłoński A, Nowak A, Zębek S, Arent Z, Bocian Ł, et al First overall report of Leptospira infections in wild boars in Poland. Acta Vet Scand. 2015;58: 3 10.1186/s13028-016-0186-7 PMC4710009 26754249

[16] Koizumi N, Muto M, Yamada A, Wanatabe H. Prevalence of Leptospira spp. in the kidneys of wild boars and deer in Japan. J Vet Med Sci. 2009;71: 797–799. 19578291 10.1292/jvms.71.797

[17] Koizumi N, Muto M, Yamamoto S, Baba Y, Kudo M, Tamae Y, et al. Investigation of reservoir animals of Leptospira in the northern part of Miyazaki Prefecture. Jpn J Infect Dis. 2008;61: 465–468. 19050356

[18] Jansen A, Luge E, Guerra B, Wittschen P, Gruber AD, Loddenkemper C, et al. Leptospirosis in urban wild boars, Berlin, Germany. Emerg Infect Dis. 2007;13: 739–742. 10.3201/eid1305.061302 17553254 PMC2738438

[19] Aviat F, Blanchard B, Michel V, Blanchet B, Branger C, Hars J, et al. Leptospira exposure in the human environment in France: A survey in feral rodents and in fresh water. Comp Immunol Microbiol Infect Dis. 2009;32: 463–476. 10.1016/j.cimid.2008.05.004 18639932

[20] Michel V, Ruvoen-Clouet N, Menard A, Sonrier C, Fillonneau C, Rakotovao F, et al. Role of the coypu (Myocastor coypus) in the epidemiology of leptospirosis in domestic animals and humans in France. Eur J Epidemiol. 2001;17: 111–121. 10.1023/A:1017931607318 11599683

[21] Mérien F, Amouriaux P, Perolat P, Baranton G, Saint Girons I. 1992. Polymerase chain reaction for detection of Leptospira spp. in clinical samples. J Clin Microbiol. 1992;30: 2219–2224. 1400983 10.1128/jcm.30.9.2219-2224.1992 PMC265482

[22] Zilber AL, Picardeau M, Ayral F, Artois M, Demont P, Kodjo A, et al. High-resolution typing of Leptospira interrogans strains by multispacer sequence typing. J Clin Microbiol. 2014;52: 564–571. 10.1128/JCM.02482-13 24478489 PMC3911330

[23] Salaün L, Mérien F, Gurianova S, Baranton G, Picardeau M. Application of multilocus variable-number tandem-repeat analysis for molecular typing of the agent of leptospirosis. J Clin Microbiol. 2006;44: 3954–3962. 10.1128/JCM.00336-06 17088367 PMC1698352

[24] Clopper CJ, Pearson ES. The use of confidence or fiducial limits illustrated in the case of the binomial. Biometrika. 1934;26: 404–413. 10.1093/biomet/26.4.404

[25] Bourhy P, Bremont S, Zinini F, Giry C, Picardeau M. 2011 Comparison of real-time PCR assays for detection of pathogenic Leptospira spp. in blood and identification of variations in target sequences. J Clin Microbiol. 2011; 49: 2154–2160. Available at: 10.1128/JCM.02452-10 21471336 PMC3122738

[26] Baradel JM, Barrat J, Blancou J, Boutin JM, Chastel C, Dannacher G, et al. [Results of a serological survey of wild mammals in France]. Rev Sci Tech OIE. 1988;7: 861–883. 10.20506/rst.7.4.375 32370370

[27] Hernández-Rodríguez P, Díaz CA, Dalmau EA, Quintero GM. A comparison between polymerase chain reaction (PCR) and traditional techniques for the diagnosis of leptospirosis in bovines. J Microbiol Methods. 2011;84: 1–7. 10.1016/j.mimet.2010.10.021 21047532

[28] Viana M, Mancy R, Biek R, Cleaveland S, Cross PC, Lloyd-Smith JO, et al. Assembling evidence for identifying reservoirs of infection. Trends Ecol Evol. 2014;29: 270–279. 10.1016/j.tree.2014.03.002 24726345 PMC4007595

[29] Mailloux M. Le hérisson vecteur de leptospiroses dans l’Ouest de la France. Med Mal. Inf. 1973;3–4: 175.

[30] Fennestad KL, Borg-Petersen C. Leptospirosis in Danish wild mammals. J Wildlife Dis. 1972;8: 343–351. 10.7589/0090-3558-8.4.343 4564183

[31] Wolf JW, Bohlander MJ. Leptospiral infection of hedgehogs in the Netherlands. Trop Geo Med. 1965;9: 9–16. 14317232

[32] Babudieri B, Farina R. The Leptospirae of the Italian hedgehog. Path Microbiol. 1964;27: 103–116. 14107351 10.1159/000161457

[33] Broom JC, Coghlan J, Kmety E. Leptospira Bratislava isolated from a hedgehog in Scotland. Lancet. 1960;275: 1326–1327. 10.1016/S0140-6736(60)92306-0 13804801

[34] Jansen A, Schöneberg I, Frank C, Alpers K, Schneider T, Stark K. Leptospirosis in Germany, 1962–2003. Emerg Infect Dis. 2005;11: 1048–1054. 10.3201/eid1107.041172 16022779 PMC3371786

[35] Meites E, Jay MT, Deresinski S, Shieh W-J, Zaki SR, Tompkins L, et al. Reemerging leptospirosis, California. Emerg Infect Dis. 2004;10: 406–412. 10.3201/eid1003.030431 15109405 PMC3322787

[36] Abgueguen P, Delbos V, Blanvillain J, Chennebault JM, Cottin J, Fanello S, et al Clinical aspects and prognostic factors of leptospirosis in adults. Retrospective study in France. J Infect. 2008;57: 171–178. 10.1016/j.jinf.2008.06.010 18656263

[37] Sharma S, Vijayachari P, Sugunan AP, Natarajaseenivasan K, Sehgal SC. Seroprevalence of leptospirosis among high-risk population of Andaman Islands, India. Am J Trop Med Hyg. 2006;74: 278–283. 16474084

[38] Deutz A, Fuchs K, Schuller W, Nowotny N, Auer H, Aspöck H, et al [Seroepidemiological studies of zoonotic infections in hunters in southeastern Austria—prevalences, risk factors, and preventive methods]. Berl Munch Tierarztl Wochenschr. 2003;116: 306–311. 12894685

