# Supplementary figures and images for: Hedgehogs and Mustelid Species: Major Carriers of Pathogenic Leptospira, a Survey in 28 Animal Species in France (20122015)
Source: PLoS One. 2016 Sep 28;11(9):e0162549. doi: 10.1371/journal.pone.0162549 (PMC5040419; doi:10.1371/journal.pone.0162549)

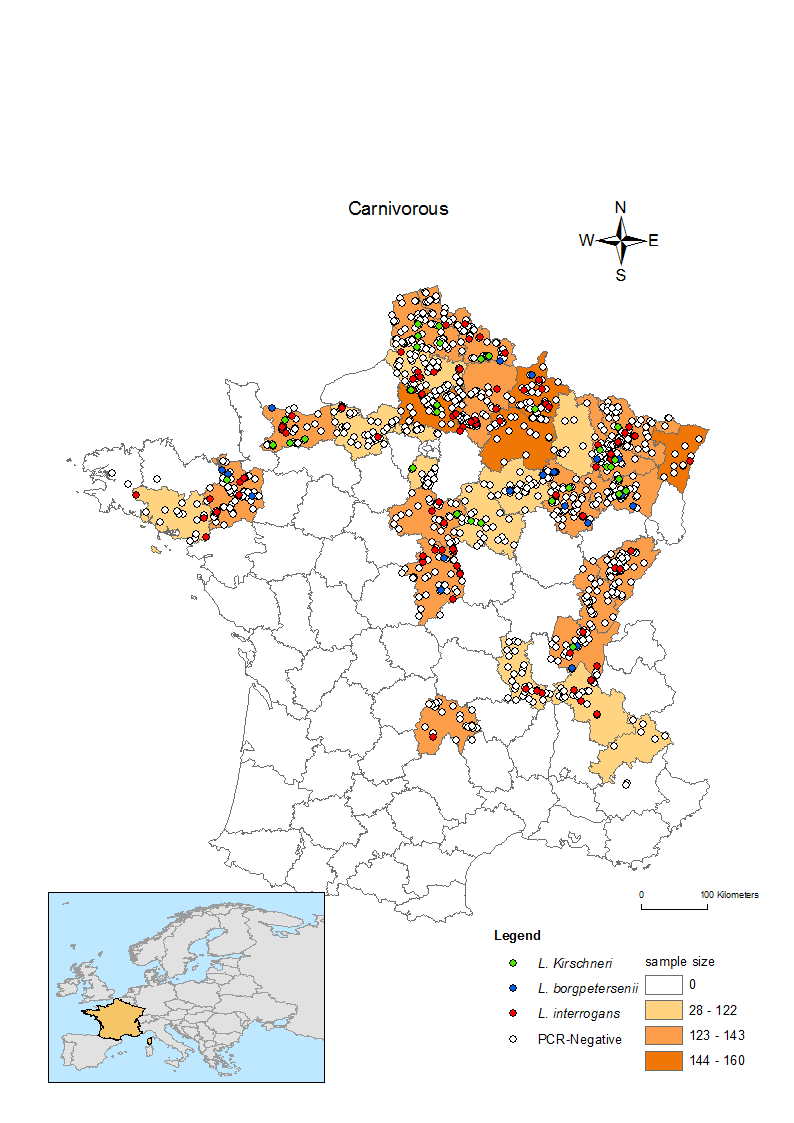

Supplement: S1 Fig — (TIF) [file pone.0162549.s001.tif]

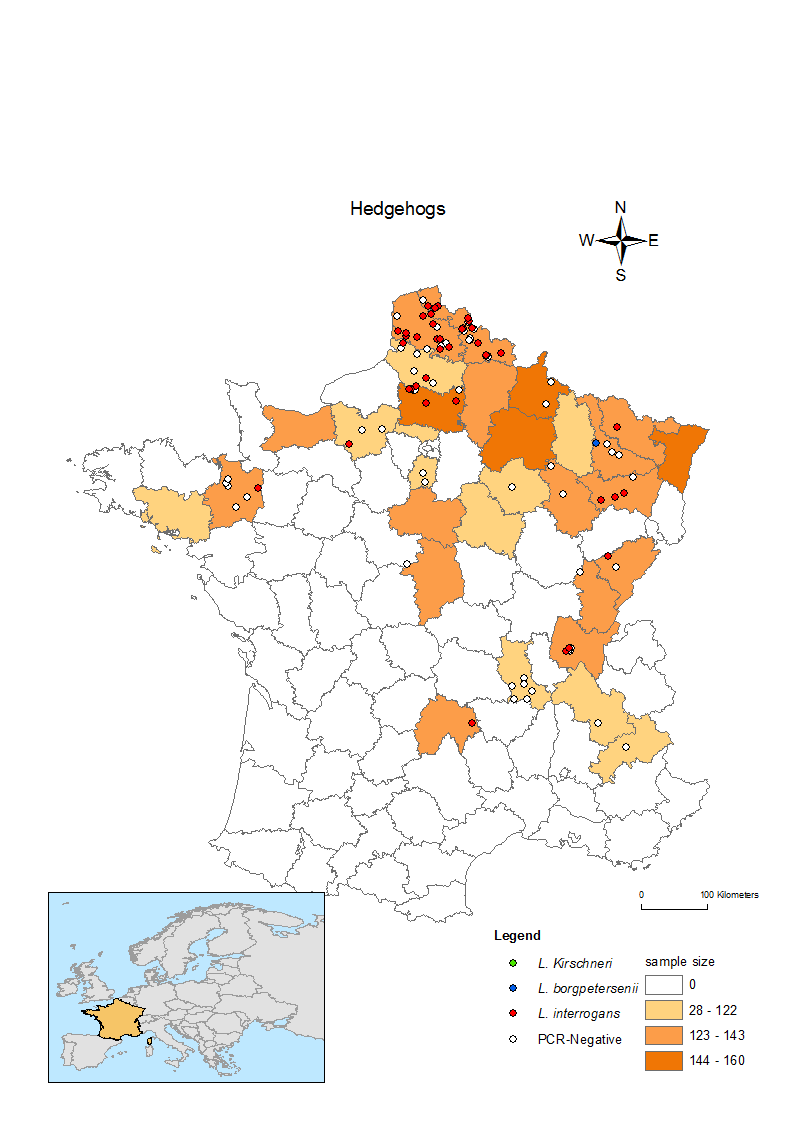

Supplement: S2 Fig — (TIF) [file pone.0162549.s002.tif]
